# Favorable Pregnancy Outcome in a Dicavitary Twin Gestation: A Case Report and Literature Review

**DOI:** 10.1155/crog/1477270

**Published:** 2026-06-08

**Authors:** Sara N. Peters, Emily J. Bollinger, Stacey S. Schmiedecke, Julie A. Watters

**Affiliations:** ^1^ OB/GYN Department, Naval Medical Center San Diego, San Diego, California, USA, nmcsd.med.navy.mil

**Keywords:** complicated twin pregnancy, dicavitary twins, didelphys uterus, Müllerian anomaly, twin gestation, uterine didelphys

## Abstract

Congenital Müllerian anomalies pose unique risks for complications in pregnancy, particularly in the setting of multifetal gestation. Uterine didelphys with dicavitary twin gestation remains a rare entity with fewer than 10 cases reported in the medical literature. Currently, there are no established guidelines for the management or delivery of dicavitary twin gestations. We report a case of naturally conceived dicavitary twins in the setting of uterine didelphys with two functional cervices and a prior vaginal septum excised in childhood. Delivery occurred in the 35th week of gestation via cesarean section with two low vertical hysterotomy incisions. Late‐preterm delivery was recommended for maternal indications, specifically superimposed preeclampsia with severe features. Cesarean delivery was recommended due to malpresentation, with both fetuses in a double‐footling breech position. The patient and twins were discharged home in good condition on Postpartum Day 3 (Day of Life 4). This case contributes to the literature on the management of a rare obstetric condition with favorable outcomes for the mother and infants, including a nuanced surgical technique.

## 1. Introduction

Congenital uterine anomalies (CUA) consist of various malformations of the uterus that arise during embryologic development of the female genitalia. The exact prevalence of CUA in the general population is not known but is estimated to be between 3.5% and 13%, and uterine didelphys accounts for approximately 8% of CUA [1]. Uterine didelphys is defined as failure of fusion of the bilateral Müllerian ducts resulting in two separate uterine cavities separated by a deep fundal cleft with noncommunicating endometrial cavities [2]. The cervix is duplicated, and a longitudinal or oblique vaginal septum is present in 75% of cases [3]. Congenital Müllerian anomalies pose unique risks for complications in pregnancy, particularly in the setting of multifetal gestation. Singleton pregnancies in patients with uterine didelphys have an increased rate of spontaneous abortion, preterm birth, and fetal growth restriction [1, 4, 5]. Uterine didelphys with dicavitary twin gestation remains a rare entity, with eight cases reported in the medical literature and an estimated incidence of 1 in 1,000,000 [5]. Six of the published case reports describe naturally conceived dicavitary twins; the remainder were conceived with assisted reproductive technology [6–13]. Due to the exceedingly rare incidence, there are currently no established societal guidelines for the management or delivery of dicavitary twin gestations. The current literature consists of a variety of delivery methods and serves as a reference for providers caring for patients with dicavitary twin pregnancies. The aim of this manuscript is to expand the existing literature on the management of dicavitary twin gestations resulting in favorable maternal and neonatal outcomes and to introduce a nuanced surgical technique.

## 2. Case Presentation

**Figure 1:**
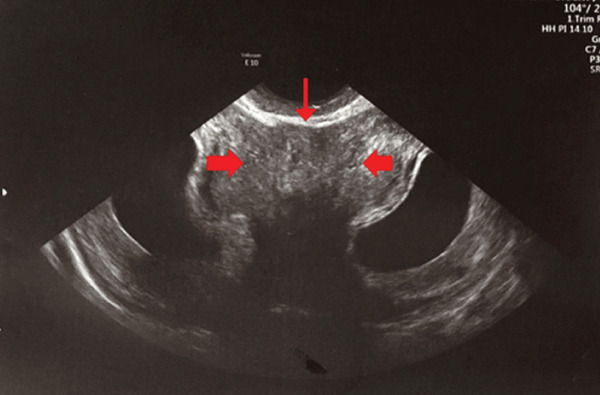
Early pregnancy ultrasound showing two separate uterine cavities separated by a fundal cleft (thin arrow), thick myometrium (thick arrows), and noncommunicating endometrial cavities, each with a sonolucent gestational sac and embryo (not shown), consistent with a dicavitary twin gestation.

A 24‐year‐old Gravida 2 Para 0 at 8 weeks of gestation presented to our medical center to establish prenatal care. She had a known history of uterine didelphys confirmed with MRI, with prior longitudinal vaginal septum excision. Prior imaging had also confirmed the presence of normal bilateral renal, ureter, and bladder anatomy. Her current pregnancy was a naturally conceived dichorionic diamniotic twin gestation with one viable embryo in each hemiuterus, diagnosed via transvaginal ultrasound at the first obstetric visit (Figure 1). Her obstetric history was notable for a 16‐week missed abortion, treated surgically with dilation and curettage 18 months prior. She also carried a diagnosis of Stage 1 chronic hypertension, not requiring antihypertensive medications but managed with low‐dose aspirin beginning at 12 weeks of gestation for preeclampsia prophylaxis. She had no other significant medical history.

The patient was comanaged with maternal–fetal medicine and underwent serial fetal ultrasound assessment starting at 13 weeks of gestation. The fetuses were both measuring consistent with dates initially; however, there was a subtle decline in the growth curve with each subsequent growth scan. At 27 weeks of gestation, Fetus A had an estimated fetal weight (EFW) in the 11th percentile, and Fetus B had an EFW in the 13th percentile. At 31 weeks of gestation, the patient developed new elevations in blood pressure, persistently in the mild range without severe elevations. Serum and urine laboratory workup noted new mild proteinuria (Table 1), not meeting criteria for superimposed preeclampsia or hemolytic anemia–elevated liver enzyme–low platelet syndrome (HELLP). She was presumed to have worsening chronic hypertension at this time due to the lack of signs or symptoms of preeclampsia. Serial laboratory assessment remained stable for the remainder of the pregnancy, but the patient’s blood pressures continued to increase.

**Table 1:** Maternal laboratory results at 31 weeks for workup of worsening chronic hypertension in pregnancy.

Laboratory test	Result	Reference range
Hemoglobin	13.7 g/dL	12.0–15.3 g/dL
Hematocrit	39.0%	36.0%–46.0%
Platelets	174,000/*μ*L	150,000–450,000/*μ*L
Creatinine	0.52 mg/dL	0.4–1.0 mg/dL
AST	32 U/L	17–63 U/L
ALT	21 U/L	12–39 U/L
Urine protein‐to‐creatinine ratio	0.15 mg/mg	< 0.30 mg/mg
Urine protein, total, 24 h	206 mg	< 300 mg

At 32 weeks of gestation, the patient was diagnosed with fetal growth restriction, with Twin A measuring at the fourth percentile and Twin B at the second percentile for gestational age. Delivery was planned for 37 weeks for severe growth restriction in Twin B, unless maternal or fetal status indicated an earlier delivery, and at this time, she began twice‐weekly antenatal testing, weekly ultrasounds for Doppler studies, and continued serial fetal growth scans. At 33 weeks of gestation, she was started on nifedipine for worsening hypertension, still not meeting criteria for superimposed preeclampsia or HELLP. Fetal status was reassuring for both twins.

**Figure 2:**
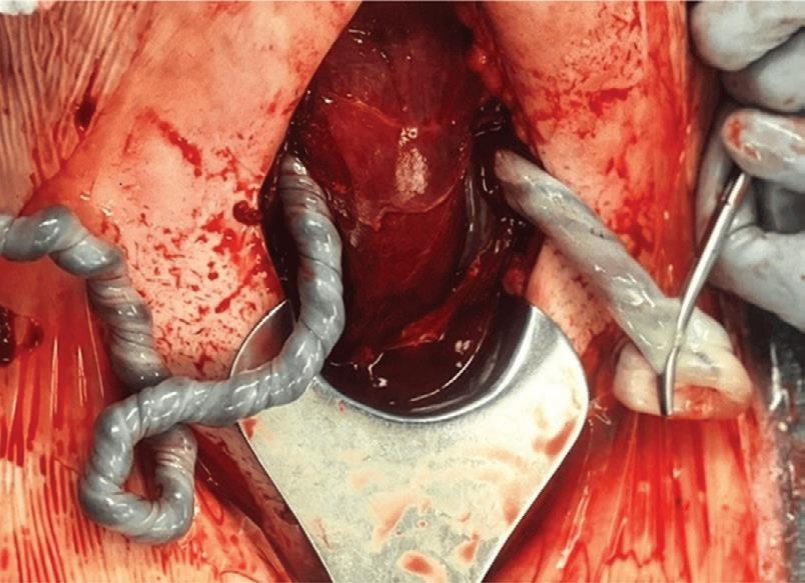
Intraoperative photograph of bilateral low vertical hysterotomies following twin delivery and cord clamping. The left uterine incision was performed first, followed by delivery of “Baby A” and delayed cord clamping per hospital protocol; the placenta was left in situ. A similar technique was used for the incision on the right uterine corpus and delivery of “Baby B.” Following delivery of the second twin, oxytocin was infused per hospital protocol, and the placentas were delivered with gentle traction on the clamped cords and massage of the uterine fundi.

**Figure 3:**
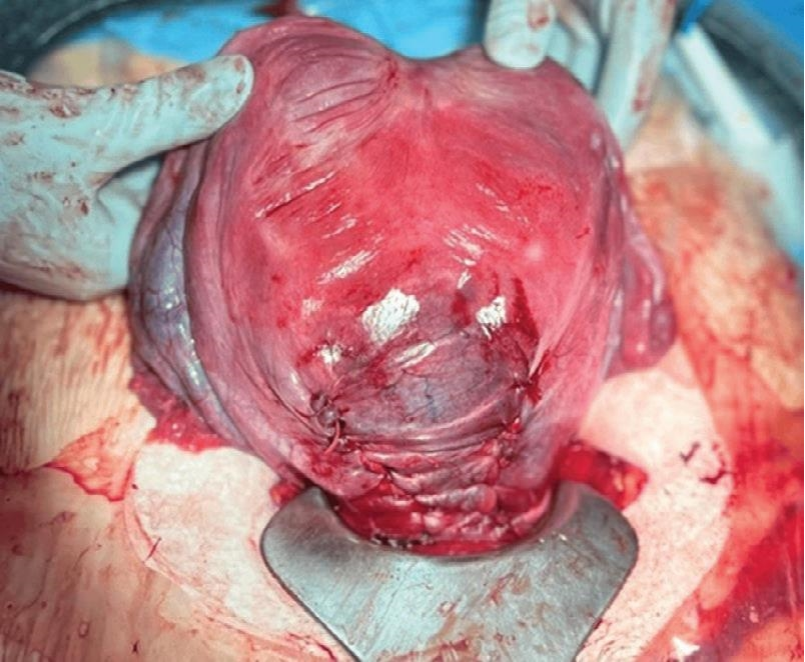
Posthysterotomy closure of the bilateral low vertical uterine incisions, each repaired in two layers.

At 35 weeks and 6 days of gestation, the patient was noted to have poorly controlled chronic hypertension with new persistent severe‐range blood pressures, despite dual antihypertensive therapy with nifedipine and labetalol. The decision was thus made to deliver for superimposed preeclampsia with severe features based on sustained severe‐range blood pressures in the setting of fetal growth restriction [14]. Prior to delivery, the patient received magnesium sulfate for maternal seizure prophylaxis and opted for betamethasone for fetal lung maturity in the late‐preterm period. Due to the double‐footling breech presentation of both twins and poorly developed lower uterine segments, delivery occurred via cesarean section with two low vertical hysterotomies (Figures 2 and 3) utilizing neuraxial anesthesia. Of note, a low transverse skin incision was performed for entry into the maternal abdomen. Hysterotomies were closed in two layers after exteriorizing the didelphys uterus. Both neonates were admitted to the neonatal intensive care unit (NICU) for brief monitoring due to prematurity and did not require respiratory or feeding support. They were subsequently discharged to the routine newborn nursery on Day of Life 2 and home on Day of Life 3 without any complications noted. The patient had an uncomplicated postpartum course and recovered appropriately. Her blood pressures improved, and antihypertensive medications were tapered over several weeks. She met postoperative milestones and was discharged with both infants in good condition on Postpartum Day 3 (Day of Life 4).

## 3. Discussion

Due to the rarity of dicavitary twin gestation, standard recommendations for management are not established. A thorough literature review revealed both vaginal and cesarean delivery for dicavitary twins, with nearly all births occurring prior to 37 weeks of gestation in these cases. The decision for the delivery route in these cases, as expected, was based on a multitude of factors, including maternal and fetal status, fetal presentations, and the presence or absence of labor. Cesarean delivery is not required, and indications for the route of delivery are similar to those of unicavitary twin gestation. In dicavitary gestations, it has been suggested that the two uterine horns contract independently of each other 90% of the time, based on limited reports of successful delayed‐interval delivery of each fetus [11, 12]. Thus, the delivery route should be based on individual factors of each fetus and corresponding unilateral maternal anatomy. While cesarean delivery with double hysterotomies is the most common route of delivery, there are cases of vaginal delivery for one fetus and cesarean delivery for the other, vaginal delivery of both fetuses, and one report of single‐hysterotomy delivery [6, 7, 9, 10]. Considerations for vaginal delivery include fetal presentations, fetal tolerance of labor, cervical changes of each cervix, and synchrony of uterine contractions between both horns.

The type of hysterotomy in a dicavitary twin gestation has also varied among reported cases, ranging from low transverse to classical incisions and even one of each [13]. The type of hysterotomy typically depends on the development of the lower uterine segment, as is routine in obstetric care, and can be different between the two uterine horns in the same patient [9]. In our patient, two low vertical hysterotomies were made due to poorly developed lower uterine segments owing to the twins’ footling breech presentations throughout the third trimester. The low vertical incisions, avoiding contractile myometrium, were documented clearly in the operative note, leaving our patient eligible for a trial of labor after cesarean delivery in future pregnancies [15].

## 4. Conclusion

We describe a rare case of dicavitary twin gestation, delivered at 35 weeks and 6 days of gestation via cesarean section with two low vertical hysterotomy incisions. For our patient, late‐preterm delivery was indicated for superimposed preeclampsia with severe features based on severe‐range blood pressures. Cesarean delivery was recommended due to malpresentation, with both fetuses in a double‐footling breech position. In cases of dicavitary twins, the indications for the route of delivery are comparable to those for a unicavitary twin gestation, taking into account fetal presentation and corresponding unilateral maternal anatomy. Our detailed literature review and summary provide information to help guide providers in their management of an exceptionally rare condition that lacks societal treatment guidelines. This case further contributes to the literature on the management, including the surgical technique, of a dicavitary twin gestation resulting in favorable outcomes for the mother and neonates.

## Funding

No funding was received for this manuscript.

## Disclosure

The views expressed are those of the authors and do not necessarily reflect the official policy or position of the Department of the Navy, DoD, or the US Government.

## Consent

Written informed consent was obtained from the participant, including consent for the images, for publication of this case report.

## Conflicts of Interest

The authors declare no conflicts of interest.

## Data Availability

Data sharing is not applicable; no new data were generated, and all referenced literature is publicly available via PubMed.
